# MicroRNA-145 in urologic tumors: biological roles, regulatory networks, and clinical translation

**DOI:** 10.3389/fphar.2025.1609646

**Published:** 2025-07-04

**Authors:** Lifeng Gan, Wei Li, Qi Chen, Le Cheng, Fangtao Zhang, Haidong Zhong, Yiran Lu, Liying Zheng, Biao Qian

**Affiliations:** ^1^ The First Clinical College, Gannan Medical University, Ganzhou, Jiangxi, China; ^2^ Department of Urology, The First Affiliated Hospital of Gannan Medical University, Ganzhou, Jiangxi, China; ^3^ Department of Urology and Andrology, The First Clinical Medical College of Gannan Medical University, The Key Laboratory of the First Clinical Medical College of Gannan Medical University, Ganzhou, Jiangxi, China; ^4^ Department of Graduate, The First Affiliated Hospital of Gannan Medical University, Ganzhou, Jiangxi, China

**Keywords:** microRNA −145, cancer, prostate cancer, bladder cancer, kidney cancer, urologic tumors

## Abstract

Tumors of the urinary system primarily encompass prostate, bladder, and kidney cancers, which exhibit high morbidity and mortality rates worldwide and pose a particularly significant threat to men’s health. Given the associated high morbidity and mortality, early diagnosis and effective treatment are crucial. Consequently, innovative research is urgently needed to enhance the clinical care of patients with urologic cancers. Recent studies have demonstrated that microRNAs (miRNAs), as key non-coding RNA molecules that regulate gene expression, play a vital regulatory role in malignant tumor development by binding to the mRNA 3′-UTR region. Large-scale genomic analyses (e.g., TCGA Pan-Cancer Atlas) reveal that over 50% of miRNA genes reside in cancer-associated regions, regulating >60% of protein-coding genes. miR-145 exemplifies this paradigm, with its dysregulation causally linked to tumor proliferation, metastasis, and therapy resistance. Among them, miR-145, as a regulatory molecule with significant anticancer properties, presents unique expression characteristics and functional mechanisms in urological tumours. In this review, we summarize the role of miR-145 in specific urological tumors, along with its downstream target molecules and cells, which may enhance our understanding of miR-145 in these cancers. In conclusion, miR-145 is a multifaceted regulator in urological oncology that has strong potential to range from non-invasive biomarker discovery to therapeutic strategies that work synergistically with conventional treatments, ultimately advancing precision medicine in prostate, bladder, and kidney cancers.

## Introduction

Prostate cancer (PC), clear cell renal cell carcinoma (ccRCC), and bladder cancer (BC) represent the most prevalent tumors of the urinary tract (Hashemi et al., 2022). Between 1990 and 2013, the cumulative incidence of kidney, bladder, and prostate cancers increased 2.5-fold globally. This rise in disease incidence has correspondingly resulted in a 1.6-fold increase in overall mortality (Dy et al., 2017). PC is among the three leading causes of cancer-related deaths in men and is one of the most frequently diagnosed cancers (Islami et al., 2021). According to Cancer Statistics 2022, PC accounts for 27% of all cancer diagnoses in men. Alarmingly, the proportion of late-stage diagnoses has escalated from 3.9% to 8.2% over the past decade (Siegel et al., 2022). Bladder cancer ranks as the second most prevalent genitourinary malignancy and was the 10th most common cancer worldwide in 2020 (Sung et al., 2021). ccRCC constitutes nearly 80% of renal cell carcinoma (RCC) subtypes and is the primary cause of renal cancer-related deaths (Bray et al., 2024). The majority of bladder cancers in the United States are diagnosed in men. As projected, the incidence of urologic cancers is likely to increase significantly due to population growth and aging. Consequently, screening and intervention for urologic cancers are expected to continue representing a substantial economic burden globally. Statistics indicate that the annual cost of treating and managing prostate, bladder, and kidney cancers in the United States is anticipated to reach $31.47 billion in 2020 (Mariotto et al., 2011). Therefore, the urgent medical necessity to identify reliable diagnostic biomarkers and develop effective treatment strategies for urologic tumors is evident.

**FIGURE 1:**
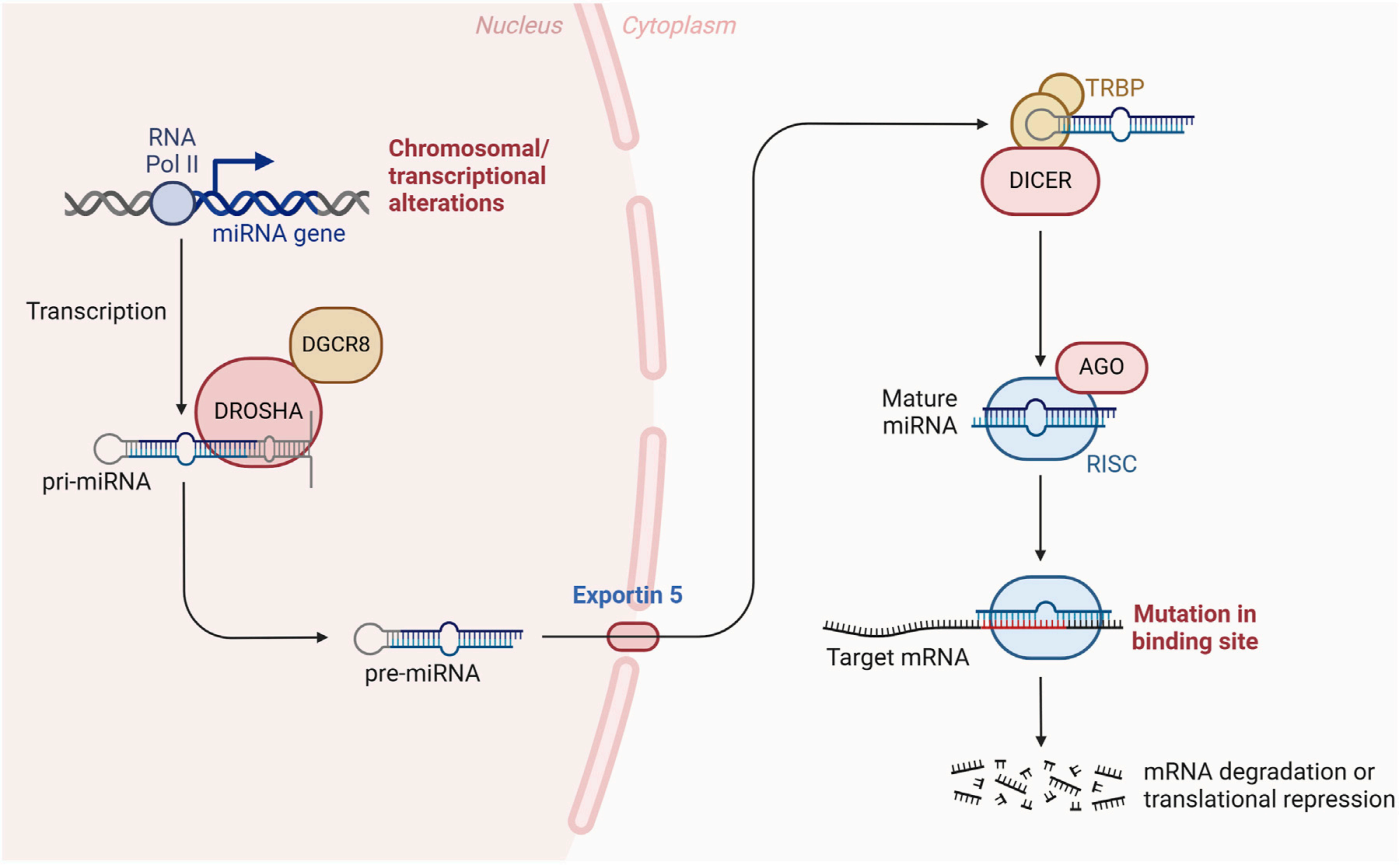
miRNA biogenesis and function. RNA polymerase II transcription generates pri-miRNA, which is sheared into pre-miRNA by the Drosha/DGCR8 complex and transported to the cytoplasm by Exportin 5; Dicer enzyme processes it into a mature miRNA duplex, which assembles into a RISC complex with the Argonaute protein to mediate degradation or translational repression via the seed sequence. Argonaute protein to assemble into a RISC complex, which targets the mRNA 3′UTR via a seed sequence to mediate degradation or translational repression. RNAPolⅡ:RNA polymerase II; DGCR8:DiGeorge syndrome critical region 8; DROSHA:Drosha Ribonuclease III; TRBP:The human TAR RNA-binding protein; AGO:Argonaute; RISC:RNA-Induced Silencing Complex.

MicroRNAs have emerged as a significant focus in both basic and translational biomedical research due to their profound influence on gene expression, their abundant presence in various body tissues and fluids, and their potential as biomarkers for disease (Yoon et al., 2020). The primary role of these small non-coding RNAs is to modulate messenger RNAs (mRNAs) by binding to recognition sites within the 3′untranslated region (UTR), thereby regulating their stability (Lee et al., 1993). Changes in miRNA expression can significantly impact the regulation of target mRNAs and, consequently, cellular homeostasis (Macfarlane and Murphy, 2010). miRNAs are traditionally produced in a sequence of events that begin in the nucleus and culminate in the cytoplasm, involving three main processes: trimming, exporting, and shearing. Initially (Kim et al., 2009; Kim, 2004; Kim, 2005; Lee et al., 2002), the miRNA gene is transcribed into the primary precursor of miRNA (pri-miRNA) by RNA polymerase II, which includes capping and polyadenylation (Lee et al., 2004). Within the nucleus, the pri-miRNA is processed into pre-miRNA by the RNase III enzyme Drosha, in conjunction with its partner DGCR8 (Lee et al., 2003; Denli et al., 2004). Following this, the pre-miRNA is transported from the nucleus to the cytoplasm via Exportin 5, where it is further processed by another RNase III enzyme, Dicer, into a mature double-stranded miRNA of 20–22 nucleotides (Hutvágner et al., 2001; Ketting et al., 2001). Upon the formation of miRNA duplexes, they associate with Argonaute (Ago) proteins to form an RNA-induced silencing complex (RISC), which subsequently directs the RISC to target mRNAs (Schwarz et al., 2003). The seed sequences of miRNAs are considered crucial for the selection of target mRNAs and are instrumental in initiating accelerated degradation and translational repression of these targets through complex mechanisms (Bartel, 2009) (Figure 1). Therefore, the identification of miRNA targets is essential for a comprehensive understanding of their functional roles. Current research mainly relies on the strategy of computational prediction combined with experimental validation to establish a reliable miRNA-mRNA regulatory network by means of integrating miRNA-mRNA co-expression data, thermodynamic properties of binding sites,and single-cell resolution analysis (Wang et al., 2021; Tian et al., 2020). For example, miR-145 is a typical functional miRNA, and its target identification will help to elucidate the molecular regulatory pathways of this miRNA in key biological processes such as cell differentiation and tumor suppression.

MiR-145 is located on chromosome 5q32-33 and spans 4.08 kb in length. It is a highly conserved sequence compared to other non-coding small RNAs. Depending on the transcription direction, the miR-145 locus can yield two transcripts: miR-145-3p (the passenger strand) and miR-145-5p (the guide strand). The miR-145-3p transcript is processed to produce a ∼22-nucleotide miRNA (miRBase database no. MIMAT0004601), while miR-145-5p generates a 23-nucleotide fragment (miRBase database number: MIMAT0000437). MiR-145 is situated in a cluster near miR-143, with both transcripts exhibiting similar functions and being considered co-transcribed (Cordes et al., 2009). As a key tumor suppressor at the 5q31 fragile site, miR-145 plays a central role in urological tumors through epigenetic dysregulation (e.g., methylation) and target gene regulation (Transgelin-2, RAB5C, IGF1, etc.). Its expression level is closely related to tumor stage, metastasis and treatment response, and has dual value as a prognostic marker and therapeutic target (Calin et al., 2004). In the future, its stage-specific regulatory network needs to be further analysed to optimize the precision treatment strategy.

miRNA-145 (miR-145) is a tumor-associated factor that is expressed in various tumors, including prostate, bladder, and renal cancers, which are the focus of this article (Kurul et al., 2019; Dip et al., 2013; Papadopoulos et al., 2016). As a tumor-associated gene, miR-145 mediates apoptosis, invasion, and metastasis of tumor cells (Huang et al., 2012; Takai et al., 2017), influences the sensitivity of these cells to chemotherapeutic agents, and regulates tumorigenesis and progression (Goto et al., 2017; Zhang and Wu, 2015; Iscaife et al., 2018). In this review, we aim to elucidate the regulatory roles and functions of miR-145 in prostate, renal cell, and bladder cancers, with a particular emphasis on dysregulated signaling pathways. We will summarize recent advances in the development of miR-145-based cancer therapies and highlight their newly described roles in carcinogenesis, as well as their potential applications in the diagnosis and treatment of urological tumors, including prostate, kidney, and bladder cancers.

**FIGURE 2:**
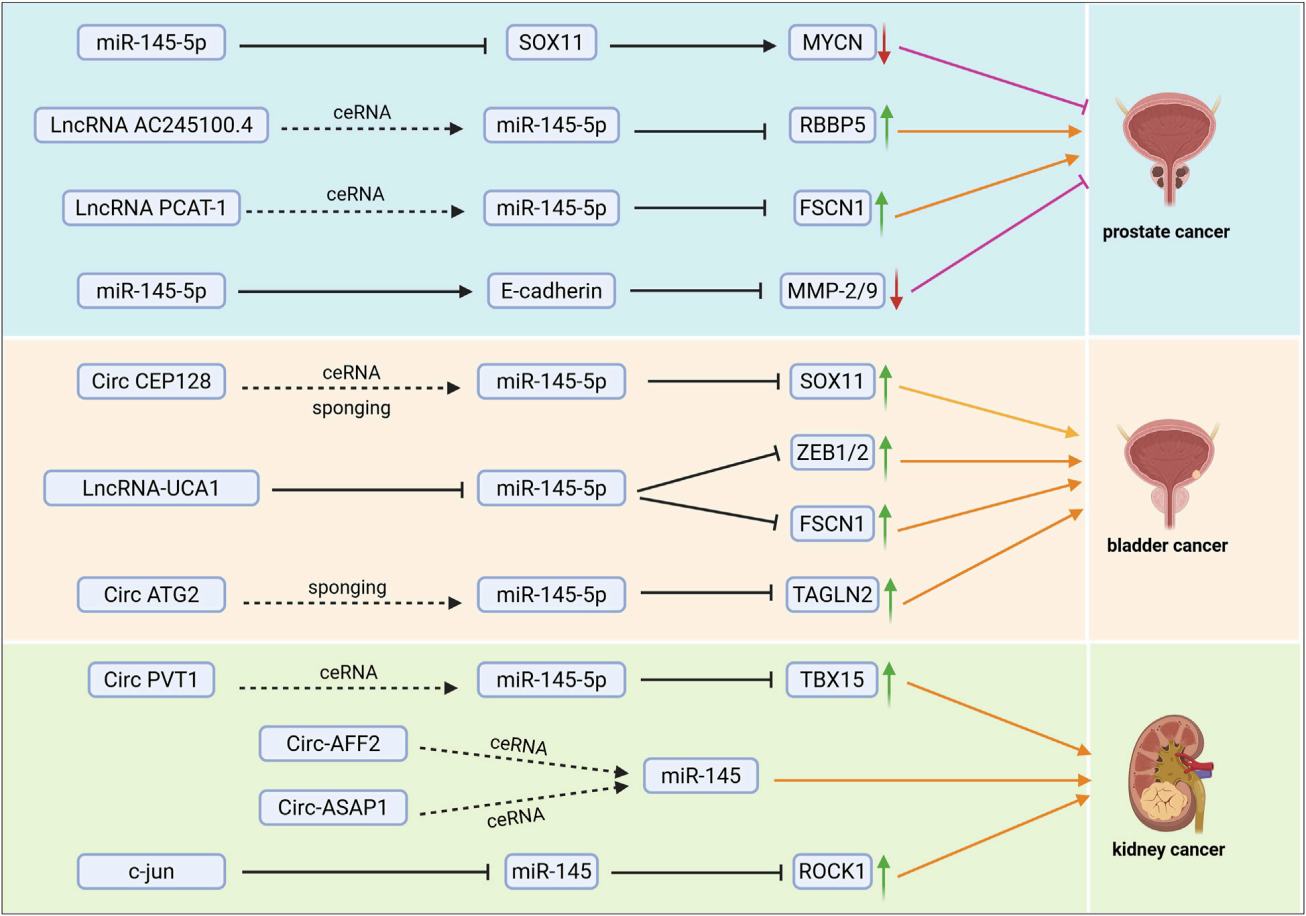
miR-145 affects the progression of urological tumours (prostate, bladder and kidney cancers) through related mechanistic pathways. ceRNA: affects miR-145 regulation of its downstream pathways by competing with miR-145 for binding to its downstream targets. Sponging: affects miR-145 binding to its downstream molecules by adsorbing miR-145. ↑: increased expression of the gene or protein; ↓: decreased expression of the gene or protein; →: promotion of the expression of downstream molecules or promotion of tumour progression; ⊣: inhibition of the expression of downstream molecules or inhibition of tumour progression.

In tumor-related studies concerning miR-145, we found that miR-145 influences tumor progression by regulating its downstream targets, while its expression in tumors can also be modulated by related genes. In prostate cancer, microRNA-145 has been identified as a crucial tumor suppressor, with its reduced expression closely linked to tumor progression and metastasis. Research indicates that miR-145 plays a significant role in the pathological processes of prostate cancer by targeting and regulating oncogene expression, inhibiting cell migration, and inducing apoptosis (Sun et al., 2021; Zhang et al., 2021). MYCN, a member of the MYC family, upregulates IFNA17 when activated in the AKT signaling pathway, thereby promoting neuroendocrine differentiation and immunosuppressive responses within the tumor microenvironment (Wen et al., 2023). Conversely, the oncogenic function of microRNA-145 may be indirectly inhibited by suppressing MYCN activity (Iscaife et al., 2018). Consequently, microRNA-145 mitigates malignant phenotypes such as metastasis and invasion in prostate cancer through dual regulation of SOX11 (impacting epithelial proteins) and MYCN (mediating neuroendocrine differentiation and epithelial-mesenchymal transition, EMT) signaling (Ji et al., 2022) (Figure 2). miR-145-5p inhibits WIP1 translation by directly binding to its mRNA 3′UTR, which blocks the activation of the WIP1-mediated PI3K/AKT signaling pathway while enhancing the phosphorylation levels of ChK2 and p-p38MAPK. This dual regulatory effect leads to significant inhibition of prostate cancer cell proliferation, invasion, and clonogenic ability, whereas overexpression of WIP1 reverses the anticancer effects of miR-145-5p, confirming that WIP1 is a core effector molecule in this pathway (Sun et al., 2021). PLD5 is highly expressed in cancer tissues and exhibits pro-oncogenic effects. Dual luciferase reporter gene assays confirmed that miR-145-5p directly inhibits PLD5 expression by binding to its 3′UTR. Functional experiments, including MTT, scratch, and Transwell assays, demonstrated that overexpression of miR-145-5p reverses the oncogenic effects of PLD5, leading to the inhibition of prostate cancer cell proliferation, migration, and invasion, as well as the induction of apoptosis. Further analysis revealed that the miR-145-5p/PLD5 axis inhibits tumor metastasis by regulating epithelial-mesenchymal transition (EMT)-related molecules, such as E-cadherin and N-cadherin (Liu et al., 2021). Notably, miR-145-5p was significantly underexpressed in metastatic prostate cancer, while TOP2A was activated during metastasis as its downstream target gene. Bioinformatics analysis, combined with experimental validation, indicated that the deletion of miR-145-5p lifted the inhibitory effect on TOP2A, resulting in the activation of the EMT-related pathway, which was evidenced by enhanced cell migration and invasion (Huang et al., 2021).

As verified by dual luciferase reporter assay, miR-145-5p inhibited the expression of SOX2 by directly targeting its 3′UTR region, which was negatively correlated with SOX2 in prostate cancer, and the mRNA and protein levels of SOX2 expression were significantly reduced in miR-145-5p overexpressing PCa cells. Functional analyses such as CCK-8 and Transwell showed that miR-145-5p overexpression affected prostate progression by inhibiting proliferation through apoptosis and reducing migration in PCa cells (Ozen et al., 2015). CDH2, a member of the calreticulin family, plays a crucial role in driving malignant phenotypes in tumor cells by promoting intercellular adhesion and activating downstream signaling pathways, such as EMT. It has been observed that the expression of miR-145 is downregulated in prostate cancer, which leads to elevated levels of CDH2 protein. This increase in CDH2 enhances cancer cell motility and invasiveness; however, these malignant phenotypes can be significantly inhibited by the exogenous restoration of miR-145 expression. Furthermore, the re-expression of CDH2 can reverse the inhibitory effects of miR-145 on cell migration and invasion (Zeng et al., 2021). The overexpression of miR-145 can be induced by directly targeting and upregulating SEMA3A, with the direct regulatory relationship between miR-145 and the SEMA3A promoter region being confirmed through chromatin immunoprecipitation (ChIP) and luciferase reporter assays. This activation of the miR-145/SEMA3A axis inhibits the proliferative capacity of prostate cancer cells and may enhance the tumor suppressor effect through a p53-dependent molecular mechanism (Barlak et al., 2021). Additionally, overexpression of metadherin (MTDH) has been associated with poor patient prognosis. Pan et al. demonstrated that MTDH overexpression promotes the viability, invasion, and migration of prostate cancer cells. They identified miR-145-5p and miR-145-3p from a set of 16 miRNAs that are closely related to prostate cancer and are targets of MTDH; both miRNAs significantly inhibit prostate cancer cell growth and metastasis by negatively regulating MTDH expression (Pan et al., 2019). It was shown that the expression of miR-145 is decreased in PC-3 cells, while the expression of SENP1, a member of the SENP family, is increased. The proliferation of PC-3 cells is inhibited by miR-145 and promoted by SENP1. Furthermore, SENP1 has been identified as a direct target of miR-145 in PC-3 cells, and the overexpression of miR-145 inhibits the proliferation of PC-3 cells induced by SENP1. Thus, overexpression of miR-145 effectively inhibits SENP1-promoted proliferation in PC-3 cells (Wang et al., 2015).

The expression of miR-145 can be regulated through mechanisms involved in the development and progression of prostate cancer, mediating its effects by influencing the regulation of downstream targets. Long non-coding RNAs (lncRNAs) AC245100.4 and retinoblastoma-binding protein 5 (RBBP5) both possess a response element for miR-145-5p. AC245100.4 adsorbs miR-145-5p, preventing its binding to RBBP5 mRNA, which leads to the upregulation of RBBP5 expression. It has been demonstrated that AC245100.4 antagonizes the inhibitory effect of miR-145-5p on RBBP5 through a competing endogenous RNA (ceRNA) mechanism, synergizing with the activation of the PAR2/p38-MAPK pathway. This interaction promotes the proliferation and migration of prostate cancer cells (Xie et al., 2021) (Figure 2). Conversely, circ_KATNAL1, which is significantly under-expressed in prostate cancer cells, functions as a ceRNA for miR-145-3p, thereby disrupting the inhibitory effect of miR-145-3p on the target gene WISP1 by directly binding to it at multiple complementary sites. Specifically, the overexpression of circ_KATNAL1 downregulates the expression of matrix metalloproteinases MMP-2 and MMP-9 via the miR-145-3p/WISP1 axis, inhibiting cell invasiveness, and activates the cleavage of caspase-3, caspase-8, caspase-9, and PARP, inducing apoptosis. Restoration of WISP1 counteracts these effects, thereby inhibiting the proliferation, invasion, and migration abilities of prostate cancer cells while promoting apoptosis (Zheng et al., 2020). Ectopic overexpression of CASC11 from the CASC gene family significantly enhances the proliferation, migration, and colony-forming ability of DU145, LNCaP, and PC3 prostate cancer (PCa) cells. This overexpression of CASC11 leads to the inhibition of miR-145 and the overexpression of IGF1R, resulting in the activation of the PI3K/AKT/mTOR signaling pathway, which influences the malignant phenotype of prostate cancer cells (Capik et al., 2021). The long non-coding RNA (lncRNA) PCAT-1 is significantly overexpressed in cancer tissues and cells, while miR-145-5p is downregulated, demonstrating a significant negative correlation. PCAT-1 disrupts the inhibitory effect of miR-145-5p on the target gene FSCN-1 (Fascin-1) by directly binding to miR-145-5p, which leads to the upregulation of FSCN-1 expression. FSCN-1 is a cytoskeletal regulatory protein whose overexpression promotes cell migration, invasion, and proliferation while inhibiting apoptosis (Xu et al., 2017). Additionally, lncRNA MALAT1 disrupts the inhibitory effect of miR-145-5p on the downstream target genes SMAD3 and TGFBR2 by competitively binding to miR-145-5p, forming a ceRNA mechanism, which promotes TGF-β1-induced EMT, thereby enhancing the migration and invasion of PCa cells (Zhang et al., 2021). The transcriptional repressor zinc finger E-box-binding homology box 2 (ZEB2) functions as an EMT activator. Dong et al. reported that ZEB2 directly represses the transcription of miR-145, a potent repressor of EMT. Conversely, ZEB2 is also a direct target of miR-145. Furthermore, their findings indicated that the downregulation of ZEB2 not only inhibited the invasion, migration, EMT, and stemness of PCa cells but also reduced the ability of PC-3 cells to invade bone *in vivo* (Ren et al., 2014) (Table 1).

**TABLE 1:** Mechanism of action of miR-145 in prostate cancer.

Mechanisms upstream	Mechanisms downstream	Upstream regulates downstream	Functional impact	Cancer cell line	Refs
miR-145-5p	SOX11	Downregulated	Inhibition of metastasis and invasion	PC-3	Ji et al. (2022)
miR-145-5p	WIP1	Downregulated	Inhibition of proliferation, invasion and metastasis	LNCaP,DU145,PC3	Sun et al. (2021)
miR-145-5p	PLD5	Downregulated	Inhibition of proliferation, migration, invasion and metastasis	PC3,LNCaP,DU-145	Liu et al. (2021)
miR-145-5p	TOP2A	Downregulated	Inhibition of metastasis and invasion	PC3	Huang et al. (2021)
miR-145-5p	SOX2	Downregulated	Inhibition of proliferation and migration	PC3,DU145,LNCaP	Ozen et al. (2015)
miR-145	CDH2	Downregulated	Inhibits EMT and affects metastasis	PC3,HEK	Zeng et al. (2021)
miR-145	SEMA3A	Upregulated	inhibit proliferation	PC3,DU145	Barlak et al. (2021)
miR-145	MTDH	Downregulated	Inhibition of growth and metastasis	PC3,LNCaP	Pan et al. (2019)
miR-145	SENP1	Downregulated	inhibit proliferation	PC-3	Wang et al. (2015)
AC245100.4/miR-145-5p	RBBP5	Upregulated	Promoting migration capacity	PC3,DU-145	Xie et al. (2021)
circ_KATNAL1/miR-145-3p	WISP1	Downregulated	Inhibits proliferation, invasion and migration, and promotes apoptosis	DU145,LNCaP,PC3	Zheng et al. (2020)
CASC11/miR-145	IGF1R	Downregulated	Promoting proliferation, colony formation and migration	DU145,LNCaP,PC3	Capik et al. (2021)
PCAT-1/miR-145-5p	FSCN1	Upregulated ed	Promotes migration, invasion and proliferation, and inhibits apoptosis	PC3,DU145,LNCap, C4-2	Xu et al. (2017)
MALAT1/MiR-145-5p	SMAD3/TGFBR2	Upregulated ed	Promote proliferation, migration and invasion of prostate cancer cells	LNCaP,CWR22Rv1	Zhang et al. (2021)
ZEB2	miR-145	Downregulated	Promotes invasion, migration, EMT and dryness	PC-3	Ren et al. (2014)
miR-145-5p	MYO6	Downregulated	Inhibition of the EMT process and inhibition of migration, invasion and metastasis	PC3,DU145	Armstrong et al. (2024)
miR-145-5p	EMT	Downregulated	Inhibits Pca bone metastasis and promotes apoptosis	PC3,22RV1	Luo et al. (2022)
CPEB1/miR-145-5p	TWIST1	Upregulated ed	Inhibition of EMT, migration and stem cell characterization	PC3,22Rv1	Rajabi et al. (2020)
BRE-AS1	miR-145-5p	Upregulated ed	Inhibits proliferation and promotes apoptosis	22Rv1	Chen et al. (2019)
miR-145	DAB2	Downregulated	Inhibition of migration and invasion	PC3	Xie et al. (2014)
miR-145	SWAP70	Upregulated ed	Inhibition of migration and invasion	PC3,DU145	Chiyomaru et al. (2011)
miR-145	FSCN1	Downregulated	Inhibition of proliferation, migration and invasion	PC3,DU145	Fuse et al. (2011)
lnc-ZNF30-3	miR-145-5p	Downregulated	Promoting proliferation and metastasis	PC3a,PNT1A,22Rv1,LNCaP,DU145	Le Hars et al. (2023)
WT-p53	miR-145	Upregulated ed	Inhibition of migration, invasion, EMT and dryness	PC-3	Ren et al. (2013)
HEF1	miR-145	Downregulated	Promotes EMT and bone invasion	PC-3	Guo et al. (2013)

The role of microRNA-145 in bladder cancer exhibits stage-specificity, with its expression level closely correlating with tumor metastatic behavior. Although miR-145 is typically considered a tumor suppressor, it may assume a pro-metastatic role in metastatic bladder cancer by modulating signaling pathways such as STAT3. This dual functional mechanism involves a dynamic balance between cell proliferation, migration, and invasion capabilities. In metastatic bladder cancer, exemplified by the T24T cell line, miR-145 upregulates FOXO1 expression by inhibiting the phosphorylation of STAT3 at the Tyr705 site, thereby disrupting its repression of FOXO1 transcriptional activity. This regulatory mechanism is cell type-specific: in non-metastatic T24 cells, miR-145 enhances FOXO1 promoter activity through STAT3 inhibition. Conversely, in metastatic T24T cells, the knockdown of STAT3 mimics the action of miR-145 by upregulating FOXO1, which significantly represses anchorage-independent growth (tumor-forming ability). It was confirmed that the specific knockdown of STAT3 shifted miR-145 from pro-oncogenic to oncogenic effects, indicating that STAT3 serves as a critical node in the functional switch of miR-145^60^. In bladder cancer, miR-145-5p expression was significantly downregulated, whereas TAGLN2 expression was abnormally elevated. It was confirmed by luciferase reporter gene assay that miR-145-5p was able to specifically bind to the 3′-UTR of TAGLN2, thus inhibiting its protein expression. Overexpression of miR-145-5p significantly inhibited the proliferation and migration ability of bladder cancer cells, as verified by MTT and Transwell assays, while high expression of TAGLN2 promoted these malignant phenotypes. Further mechanistic studies showed that overexpression of TAGLN2, a direct downstream target of miR-145-5p, counteracted the inhibitory effects of miR-145-5p on cell proliferation and migration, forming a ‘miR-145-5p/TAGLN2 regulatory axis’. This axis affects the migration ability of bladder cancer cells by regulating the dynamics of cytoskeleton-associated proteins (e.g., F-actin), and may also affect proliferation by interfering with cell cycle-related pathways (Zhang H. et al., 2018). Kou et al. found that the levels of miR-145 were reduced while PAK1 protein expression was upregulated in bladder cancer tissues. They predicted that PAK1 was a direct target of miR-145 using bioinformatics methods and confirmed that miR-145 levels were negatively correlated with PAK1 protein expression in bladder cancer through a luciferase activity assay of the 3′-untranslated region of PAK1 messenger RNA. The negative correlation between miR-145 levels and PAK1 protein expression in bladder cancer indicates that miR-145 inhibits bladder cancer cell invasion by targeting PAK1 (Kou et al., 2014). Several studies have reported that the expression level of miR-145 is significantly reduced in clinical bladder cancer samples and bladder cancer cell lines. Furthermore, luciferase assays demonstrated that miR-145 directly binds to the 3′UTR of KLF4, and that Warburg effect-related genes, such as PTBP1 and PKMs, are regulated by bladder cancer cells transfected with miR-145 or siR-KLF4. MiR-145 disrupts the Warburg effect by inhibiting the KLF4/PTBP1/PKMs pathway in bladder cancer cells, leading to significant inhibition of cell growth (Minami et al., 2017).

A database search revealed that miR-145 has a target sequence for muscle bundle homolog 1 (FSCN1) mRNA. Luciferase assays confirmed that miR-145 directly binds to FSCN1 mRNA, resulting in the inhibition of both FSCN1 mRNA and protein expression, which in turn inhibits bladder cancer cell growth, invasion, and migration (Chiyomaru et al., 2010). Additionally, other non-coding RNAs have been shown to function as competing endogenous RNAs (ceRNAs) or miRNA sponges that are involved in bladder carcinogenesis and progression by competitively binding to or sponging miR-145 (Zhu et al., 2020). LncRNA KCNQ1OT1 exhibited a negative correlation with miR-145-5p at the expression level and a positive correlation with PCBP2. Knocking down KCNQ1OT1 resulted in the release of miR-145-5p expression, which subsequently led to a decrease in PCBP2 expression. This decrease inhibited the proliferation, migration, and invasive capabilities of bladder cancer cells while promoting apoptosis. Conversely, the inhibition of either miR-145-5p or the overexpression of PCBP2 reversed the inhibitory effects of KCNQ1OT1 knockdown on the malignant phenotype (Wang et al., 2019). High expression levels of circ_0058063 released CDK6 through the adsorption of miR-145-5p, leading to an upregulation of CDK6 protein expression. This upregulation activated cell cycle-related pathways, promoting the proliferation and migration of bladder cancer cells while inhibiting apoptosis. *In vivo* experiments further confirmed that the knockdown of circ_0058063 inhibited the growth of transplanted tumors in BALB/c nude mice (Sun et al., 2019). Gao et al. identified CDCA3 as a potential target of miR-145 through bioinformatics analysis. Experimental validation revealed that CDCA3 was upregulated in BC specimens and acted as a positive regulator of BC cell proliferation and migration. Furthermore, a luciferase reporter assay confirmed that CDCA3 is indeed a target of miR-145. Additional experiments demonstrated that LINC00707 promotes the proliferation and metastasis of bladder cancer by targeting the miR-145/CDCA3 regulatory loop (Gao and Ji, 2021) (Table 2).

**TABLE 2:** Mechanism of miR-145 in bladder cancer.

Mechanisms upstream	Mechanisms downstream	Upstream regulates downstream	Functional impact	Cancer cell line	Refs
miR-145	STAT3-FOXO1	Upregulated	promote growth	T24T	Jiang et al. (2017)
miR-145-5p	TAGLN2	Downregulated	Inhibition of proliferation and migration	T24,5637,SV-HUC-1	Zhang H. et al. (2018)
miR-145	PAK1	Downregulated	Suppression of infestation	RT4,5637,T24,J82, 253J	Kou et al. (2014)
miR-145	KLF4/PTBP1/PKMs	Downregulated	cell growth inhibition	T24	Minami et al. (2017)
miR-145	FSCN1	Downregulated	Inhibition of growth, invasion and migration	T24,KK47	Chiyomaru et al. (2010)
KCNQ1OT1/miR-145	PCBP2	Upregulated	Promotes proliferation, migration and invasion, and inhibits apoptosis	UM-UC-3,T24,HT-1376,HT-1197	Wang et al. (2019)
Circ_0058063/miR-145-5p	CDK6	Downregulated	Promotes proliferation and migration and inhibits apoptosis	T24,J82	Sun et al. (2019)
LINC00707/miR-145	CDCA3	Upregulated	Promoting proliferation, colony formation and translocation	UMUC3,T24T	Gao and Ji (2021)
miR-145	N-Calmodulin	Downregulated	Inhibition of migration and invasion	T24	Zhang X. F. et al. (2018)
miR-145	IGF-IR	Downregulated	Inhibition of proliferation and migration	T24,5637	Zhu et al. (2014)
miR-145	SOCS7	Downregulated	induction of apoptosis	T24	Noguchi et al. (2013)
CircCEP128/miR-145-5p	SOX11	Upregulated	Promotes proliferation and inhibits apoptosis	RT112,5637,BIU-87,TCCSUP	Wu et al. (2018)
ATG7/autophagy/FOXO3A/miR-145	pd-l1 mRNA	Upregulated	promote aggression	T24,T24T	Zhu et al. (2019)
circSTAG2 (16–25)/miR-145	TAGLN2	Upregulated	Promoting relapse	T24,5637	Du et al. (2024)
lncRNA-UCA1/hsa-miR-145	ZEB1/2/FSCN1	Upregulated	Promoting migration and invasion	5637,T24,UMUC2	Xue et al. (2016)
HIF	miR-145	Upregulated	promote apoptosis	RT4	Blick et al. (2015)

MicroRNA-145 is widely recognized as a tumor suppressor in RCC, with its downregulation closely associated with poor patient prognosis. Studies have demonstrated that miR-145 plays a crucial regulatory role in renal cancer development by modulating pathways related to cell proliferation, invasion, and apoptosis. Its molecular mechanism involves the targeted inhibition of various oncogenes and signaling pathways (Liep et al., 2016). Circular RNA PVT1 (circPVT1), functioning as a competitive endogenous RNA, directly binds to miR-145-5p, thereby preventing it from binding to the 3′-UTR region of TBX15 mRNA through the “sponge effect.” This inhibition leads to the upregulation of TBX15 expression. The activation of the circPVT1/miR-145-5p/TBX15 axis significantly enhances the proliferative capacity and metastatic potential of ccRCC cells, correlating with poor patient prognosis (Zheng et al., 2021) (Figure 2). The transcription factor c-jun is a key regulator of cancer cell growth and metastasis (Chakraborty et al., 2015; Lamb et al., 1997). Ding et al. identified four putative binding sites for c-jun in the region upstream of the miR-145 locus. Subsequent experiments indicated that c-jun negatively regulates the expression of miR-145 by directly targeting its promoter, thus contributing to miR-145 dysregulation in ccRCC. Additionally, the overexpression of miR-145 significantly downregulates ROCK1 mRNA and protein levels in A498 renal cancer cells, inhibiting cell proliferation and invasion while inducing apoptosis (Ding et al., 2018) (Figure 2). The androgen receptor (AR) has been shown to promote the invasion and proliferation of various RCC cell lines. Specifically, the AR inhibits p53-induced miR-145 expression by binding to the miR-145 promoter through the androgen response element (ARE), located 575 bases upstream of the transcriptional start site. Additionally, it enhances RCC growth and metastasis by activating the HIF2α/VEGF/MMP9/CCND1 signaling pathway in VHL mutant SW-839 and OSRC-2 cells (Chen et al., 2015). A disintegrin and metalloproteinase 17 (ADAM17) is a metalloproteinase that also promotes RCC growth and metastasis. The HIF2α/VEGF/MMP9/CCND1 signaling in VHL mutant SW-839 and OSRC-2 cells inhibits miR-145 expression, thereby facilitating RCC growth and metastasis (Zheng et al., 2007; Tanaka et al., 2005; Ringel et al., 2006; Roemer et al., 2004). ADAM17 is overexpressed in various cancer types, including renal cancer. In RCC, ADAM17 expression correlates with increasing malignancy and is essential for the formation of xenograft tumors (Franovic et al., 2006). It has been demonstrated that miR-145 downregulates ADAM17 protein expression by directly targeting the 3′untranslated region (3′UTR) of ADAM17 mRNA, thereby reducing the proliferation and invasion of renal cancer cells. Furthermore, ADAM17 negatively regulates miR-145 expression through TNF-α, creating a reciprocal negative feedback loop (Doberstein et al., 2013) (Table 3).

**TABLE 3:** Mechanism of miR-145 in renal cancer.

Mechanisms upstream	Mechanisms downstream	Upstream regulates downstream	Functional impact	Cancer cell line	Refs
circPVT1/miR-145-5p	TBX15	Upregulated	Promoting proliferation and metastasis	786-O,A498	Zheng et al. (2021)
c-jun/miR-145	ROCK1	Upregulated	Promotes proliferation and invasion and inhibits apoptosis	A498	Ding et al. (2018)
AR/miR-145	HIF2α/VEGF/MMP9/CCND1	Upregulated	Promotes proliferation and invasion and inhibits apoptosis	SW-839,ACHN,HK2	Chen et al. (2015)
miR-145	ADAM17	Downregulated	Inhibition of proliferation and invasion	A498	Doberstein et al. (2013)
circ-AFF2,circ-ASAP1	miR-145	Upregulated	promotion of proliferation	ACHN,786-O	Ji et al. (2023)
miR-145	MMP-11	Downregulated	Inhibition of proliferation, migration and invasion	786-O,A498	Wu et al. (2014)

### MiR-145 regulates core hallmarks of urologic tumors

miR-145 plays a crucial regulatory role in various tumors, participating in biological processes such as tumor cell proliferation, apoptosis, metastasis, invasion, EMT, and stemness regulation by targeting oncogenes and signaling pathways. In urological tumors, the level of miR-145 is closely associated with tumor malignancy, clinical staging, and patient prognosis, indicating that miR-145 may serve as a significant target for the diagnosis and treatment of urological tumors. This paper will systematically describe the biological properties of miR-145 in urological tumors through three dimensions: proliferation and apoptosis, metastasis and invasion, and EMT and stemness in these three types of cancers.

### Proliferation and apoptosis

miR-145 significantly inhibits aberrant proliferation and induces programmed cell death in urological tumor cells by targeting key regulators of the cell cycle and apoptosis-related pathways. This paragraph will explore how miR-145 balances tumor cell proliferation and apoptosis through molecular networks, highlighting common mechanisms and specific targets across various urological tumors. Studies have demonstrated that the overexpression of BRE-AS1 inhibits the proliferation of PC cells while promoting their apoptosis. Furthermore, the application of a miR-145-5p inhibitor can reverse the inhibitory effects of BRE-AS1 overexpression on cancer cell behavior, confirming that the regulatory function of BRE-AS1 relies on its molecular interactions with miR-145-5p. This indicates that BRE-AS1 may function as a ceRNA to bind miR-145-5p, thereby disrupting the inhibitory effects of miR-145-5p on downstream cancer-promoting target genes, ultimately affecting the malignant phenotype of prostate cancer (Chen et al., 2019). Additionally, high expression levels of the lncRNA PCAT-1 sequester miR-145-5p, preventing it from binding effectively to the 3′untranslated region (UTR) of FSCN1 mRNA, thus deregulating the activity of miR-145-5p. The aberrant upregulation of FSCN1 protein consequently promotes the proliferation of prostate cancer cells while inhibiting apoptosis. In bladder cancer, circCEP128 is significantly upregulated, whereas miR-145-5p is underexpressed (Xu et al., 2017). CircCEP128 directly binds to and “sponges” FSCN1 mRNAs through the ceRNA mechanism, and it is not expressed in the 3′UTR of FSCN1 mRNA. Moreover, circCEP128 directly binds and “sponges” miR-145-5p, thereby alleviating the inhibitory effect of miR-145-5p on its target gene SOX11. This interaction leads to the upregulation of SOX11 expression, which in turn promotes the proliferation of bladder cancer cells and inhibits apoptosis (Wu et al., 2018) (Figure 2). MiR-145 has been identified as a direct target gene of the hypoxia-inducible factor HIF-1α, with hypoxia-induced expression of miR-145 resulting from HIF-1α-dependent trans-activation. Transfection with anti-miR-145 was found to enhance cell viability under hypoxic conditions, while the upregulation of miR-145 under hypoxia led to increased apoptosis in the bladder cancer cell line RT4 (Blick et al., 2015).

**FIGURE 3:**
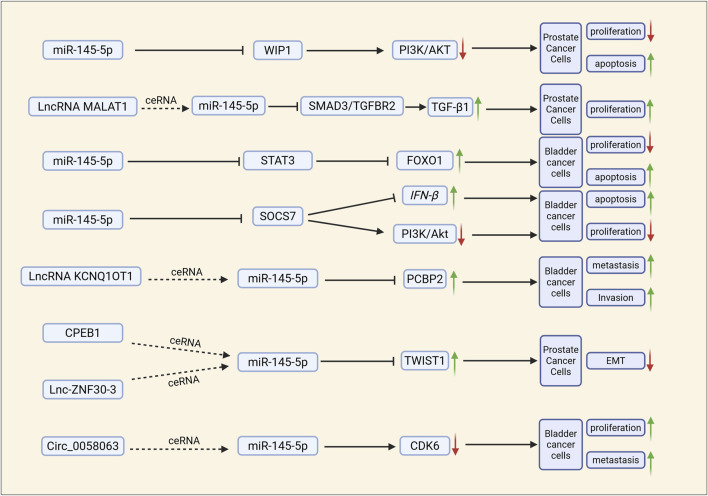
miR-145 affects the biological properties of urological tumours through related mechanistic pathways. ceRNA: affects miR-145 regulation of its downstream pathways by competing with miR-145 for binding to its downstream targets. ↑: protein expression of the gene is elevated or tumour biology is promoted; ↓: protein expression of the gene is reduced or tumour biology is inhibited; →: expression of downstream molecules is promoted; ⊣: expression of downstream molecules is inhibited.

The insulin-like growth factor receptor I (IGF-IR) is a proto-oncogene known for its potent pro-mitotic and anti-apoptotic properties. Studies have reported an upregulation of IGF-IR expression in bladder cancer, and it has been demonstrated that miR-145 directly targets the 3′-untranslated region (UTR) of IGF-IR in human bladder cancer cells. Knockdown assays utilizing small interfering RNA (siRNA) and miR-145 confirmed that miR-145 promotes apoptosis and inhibits cell proliferation by downregulating IGF-IR expression (Zhu et al., 2014). Exogenous miR-145 significantly enhances the expression levels of interferon (IFN)-β, 2′-5′-oligoadenylate synthetase 1 (which is upstream of the 2′-5′ oligoadenylate/RNase L system), and TRAIL in bladder cancer T24 and NKB1 cells, leading to apoptotic cell death in human bladder cancer cells. miR-145 enhances IFN-β expression and facilitates IFN-IR knockdown in these cells by targeting socs7, while also inhibiting cell proliferation through the downregulation of IGF-IR expression. Additionally, miR-145 promotes apoptosis and suppresses cell proliferation via the inhibition of IGF-IR. The self-induction of IFN-β, achieved by targeting socs7, results in the nuclear translocation of STAT3, whereas SOCS7 promotes bladder cancer cell growth by activating the PI3K/Akt signaling pathway (Noguchi et al., 2013) (Figure 3). Circ-AFF2 and circ-ASAP1, which are upregulated in RCC, act as molecular sponges by competitively binding to miR-145. These two circRNAs impede the inhibitory effect of miR-145 on downstream oncogenes by adsorbing miR-145, thereby neutralizing the tumor suppressor function of miR-145. This interaction culminates in enhanced proliferation, reduced apoptosis, and an increased metastatic tendency of RCC cells, ultimately facilitating tumor progression (Ji et al., 2023).

### Metastasis and invasion

Metastasis and invasion are the primary causes of mortality in patients with urological tumors, and miR-145 significantly diminishes the metastatic potential of tumor cells by inhibiting EMT pathways and molecules associated with cell motility. This paragraph systematically explores how miR-145 inhibits the distant dissemination of tumors by regulating metastasis-related signaling axes and mechanisms of cytoskeletal remodeling. One study identified DAB2 (Disabled homolog 2) as a downstream target gene of miR-145 during tumor metastasis. In this study, a relatively high level of DAB2 gene expression was observed in PC3 cells, which are associated with highly aggressive prostate cancer. Moreover, the forced expression of miR-145 significantly downregulated DAB2 expression in PC3 cells and inhibited their migration and invasion (Xie et al., 2014). Investigations using miR-145 and luciferase reporter gene assays indicated that the actin-binding protein SWAP70 is directly regulated by miR-145. SWAP70 promotes cell migration and invasion in prostate cancer (CaP) cell lines. miR-145 functions by inhibiting oncogenic SWAP70 in CaP, and the knockdown of SWAP70 results in reduced migration and invasion activity of CaP cells (Chiyomaru et al., 2011). Additionally, fSCN1, another actin-binding protein, has been identified as a candidate target gene for miR-145 based on genome-wide gene expression analysis. Luciferase reporter gene assays demonstrated that the signals from two miR-145 target sites at the 3′UTR of FSCN1 were significantly attenuated, suggesting that miR-145 directly regulates FSCN1. Consequently, miR-145 inhibits cell proliferation, migration, and invasion in prostate cancer by targeting FSCN1 (Fuse et al., 2011).

CircSTAG2 binds to miR-145-5p via sponge adsorption, thereby derepressing the inhibitory effect of miR-145-5p on the target gene Transgelin 2 (TAGLN2). As a tumor suppressor, miR-145-5p directly targets and downregulates the expression of TAGLN2, a protein known to promote the motility and invasiveness of bladder cancer cells. Overexpression of circSTAG2 blocks the interaction between miR-145-5p and TAGLN2 mRNA by competitively binding to miR-145-5p, resulting in the upregulation of TAGLN2 protein expression. This upregulation activates downstream signaling pathways, ultimately enhancing the migratory and invasive malignancy of bladder cancer cells (Du et al., 2024) (Figure 2). Notably, microRNA-145 is significantly downregulated in bladder cancer, leading to a marked reduction in the expression of N-cadherin and its downstream effector molecule matrix metalloproteinase-9 (MMP9). This occurs through the direct targeting of the 3′UTR of N-cadherin, thereby inhibiting its protein expression level. By impairing the N-cadherin-MMP9 pathway, miR-145 diminishes the migration and invasion capabilities of bladder cancer cells (Zhang X. F. et al., 2018). Furthermore, the non-coding RNA uroepithelial carcinoma associated 1 (lncRNA-UCA1) is highly expressed in bladder cancer tissues and cells. lncRNA-UCA1 induces EMT in bladder cancer cells by up-regulating the expression levels of zinc-finger E-box-binding homology boxes 1 and 2 (ZEB1 and ZEB2), and it regulates bladder cancer cell EMT through the tumor suppressor hsa-miR-145 and its target gene FSCN1. FSCN1 plays a critical role in regulating the migration and invasion of bladder cancer cells (Xue et al., 2016) (Figure 2). Wu et al. demonstrated that miR-145 inhibited the proliferation, migration, and invasion of renal carcinoma 786-O and A498 cells, further revealing that miR-145 suppresses these processes by directly targeting matrix metallopeptidase-11 (MMP-11) in RCC (Wu et al., 2014).

### EMT and stemness

EMT and the properties of tumor stem cells (CSCs) are pivotal mechanisms that drive malignant tumor progression. MiR-145 has been shown to reverse the mesenchymal phenotype and stem cell characteristics of tumor cells by directly targeting EMT transcription factors and stemness-related markers. This paragraph elucidates how miR-145 influences tumor progression and recurrence by inhibiting EMT processes and diminishing tumor stemness through epigenetic and post-transcriptional regulatory networks. Notably, MYO6, identified as an EMT-related gene, is a novel target of miR-145-5p, showing a negative correlation at the expression level. miR-145-5p represses MYO6 protein expression by binding to its 3′untranslated region, leading to the downregulation of EMT-related markers (e.g., vimentin, N-cadherin) and the upregulation of E-cadherin, thereby obstructing the EMT process. Functional experiments demonstrated that overexpression of miR-145-5p significantly inhibited the proliferation, colony formation, and migratory capacity of prostate cancer cells, whereas knockdown of miR-145-5p enhanced these malignant phenotypes (Armstrong et al., 2024). Furthermore, miR-145-5p interacts with lnc-ZNF30-3 in prostate cancer through a ceRNA mechanism: lnc-ZNF30-3 acts as a molecular sponge, sequestering miR-145-5p and negating its inhibitory effects on TWIST1 and other EMT-associated transcription factors. As a repressor of EMT and stemness phenotypes, the binding of miR-145-5p in large quantities to lnc-ZNF30-3 diminishes its activity, resulting in the upregulation of TWIST1 and other pro-EMT factors, which subsequently activates EMT and enhances tumor stemness, thereby promoting the migration, invasion, and metastasis of prostate cancer cells (Le Hars et al., 2023). One study demonstrated that the upregulation of miR-145-5p increased the expression of E-cadherin, an epithelial marker, while decreasing the expression of MMP-2 and MMP-9, which are mesenchymal markers, as assessed through protein blotting. miR-145-5p appears to enhance the expression of epithelial markers (E-cadherin) and suppress the expression of mesenchymal markers (MMP-2 and MMP-9). This regulation by miR-145-5p contributes to the reduction of invasion and migration by modulating EMT (Luo et al., 2022).

TWIST1, a basic helix-loop-helix transcription factor, is one of the primary regulators of EMT. miR-145-5p serves as a significant factor controlling the expression of TWIST1 in prostate cancer and is implicated in the regulation of cytoplasmic polyadenylation element-binding proteins. In CPEB1-restricted PC3 and PNT1A cells, the downregulation of miR-145-5p resulted in the upregulation of the mesenchymal marker waveform protein and the downregulation of the epithelial marker E-calmodulin, in conjunction with the accumulation of the TWIST1 EMT transcription factor (Rajabi et al., 2020). Additionally, it was observed that WT-p53 enhanced miR-145 expression in PC-3 cells, and that anti-miR-145 reversed the EMT signature of PC-3 cells that was suppressed by ectopic expression of WT-p53. These findings suggest that miR-145 acts as a mediator of WT-p53-regulated EMT (Ren et al., 2013). Guo et al. identified HEF1 as a direct target of miR-145 by constructing a luciferase reporter gene system. They demonstrated that Human Enhancer of Filamentation Protein 1 (HEF1) promotes EMT and bone invasion in prostate cancer by directly targeting miR-145, which partially inhibits EMT and invasion by downregulating HEF1 (Guo et al., 2013). A related study on bladder cancer showed that overexpression of autophagy-associated gene 7 (ATG7) impairs FOXO3a protein expression, thereby reducing the transcription of the miR-145 promoter and its overall expression through direct binding to its promoter region. Consequently, the downregulation of miR-145 diminishes its binding to the 3′-UTR of PD-L1 mRNA, resulting in mRNA stabilization and increased PD-L1 protein expression. This process further promotes stem-like properties and invasion in human bladder cancer cells. Additionally, PD-L1 was found to form a positive feedback loop that enhances the oncogenic role of ATG7 in promoting stem cell-like properties, invasion, and tumorigenesis in human bladder cancer (Zhu et al., 2019).

### Advances in miR-145 and immune checkpoint regulation and nanodelivery systems

In recent years, miR-145 has been identified as a significant player in urological tumors, not only by directly regulating oncogenic signaling pathways but also by influencing the tumor microenvironment through the modulation of immune checkpoint molecules, such as PD-L1. In bladder cancer, PD-L1 is predominantly expressed in tumor-infiltrating immune cells rather than in the tumor cells themselves, and its expression level shows a significant correlation with high-grade T1 stage tumor progression (Dallos and Drake, 2018; Wankowicz et al., 2017). Notably, miR-145 may disrupt the tumor immune escape mechanism by targeting and inhibiting PD-L1 expression. For instance, cisplatin-induced downregulation of miR-145 has been shown to significantly increase PD-L1 expression in an ovarian cancer model, a mechanism that may also contribute to chemotherapy resistance in urological tumors (Sheng et al., 2020). Additionally, PD-L1 has been significantly correlated with immunosuppressive cells, such as regulatory T cells and myeloid-derived suppressor cells, within the tumor microenvironment of renal and prostate cancers. This suggests that miR-145 may influence the function of the PD-1/PD-L1 axis by modulating these immune cell subpopulations (Liu et al., 2024; Lu et al., 2022).

In terms of delivery systems, nanotechnology-based miR-145-targeted therapies have demonstrated significant potential for clinical applications. The research team developed a novel miR-145S1 intravesical bladder delivery formulation that markedly inhibited tumor progression in a non-muscle invasive bladder cancer model. Its mechanism of action involved the restoration of the regulatory function of miR-145 on tumor stem cell differentiation (Heishima et al., 2023). Furthermore, hyaluronic acid-modified nanocarriers significantly enhanced the delivery efficiency of miR-145 in colon cancer cells, induced cell cycle arrest, and inhibited proliferation. This targeted delivery strategy provides a technological reference for the local treatment of urological tumors, particularly prostate and renal cancers (Liang et al., 2016). Notably, gold nanoplatforms (GNPF), as non-viral vectors, can be efficiently loaded with miR-145, leading to a significant inhibition of tumor growth by suppressing the PI3K/AKT signaling pathway. This delivery system has exhibited dose-dependent anti-tumor effects in prostate cancer xenograft models (Salas-Huenuleo et al., 2022; Ye et al., 2019). These breakthroughs establish an important foundation for the development of urological tumor-specific miR-145 nano-formulations.

#### MiR-145 in chemotherapy and radiotherapy for urologic tumors

**FIGURE 4:**
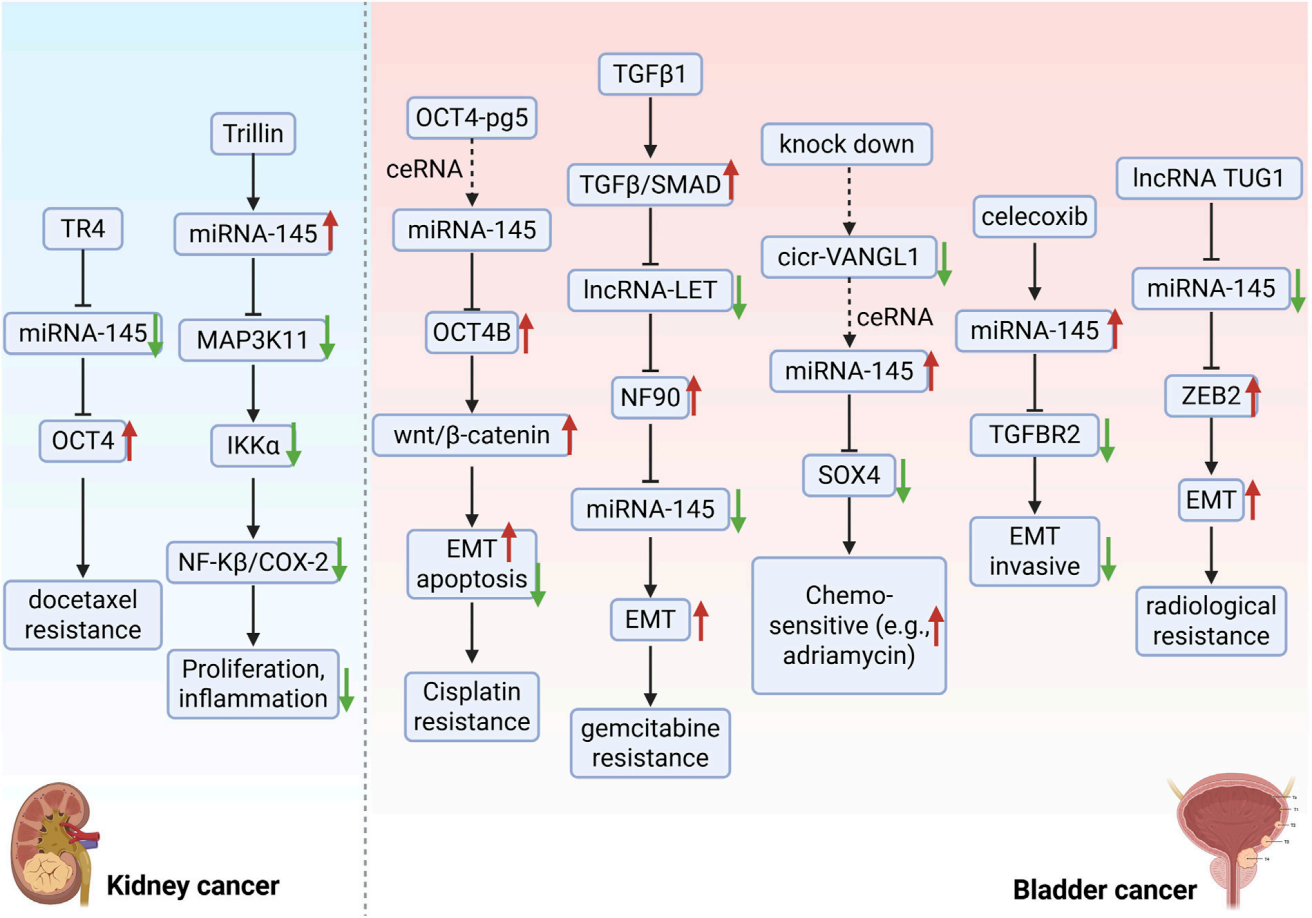
miR-145 counteracts therapy resistance through multi-target interventions. The natural compound Trillin upregulates miR-145 expression, suppresses the MAP3K11/NF-κB inflammatory pathway, and enhances treatment sensitivity in castration-resistant prostate cancer (CRPC). Celecoxib synergizes with miR-145 to block TGF-β signaling, inhibiting epithelial-mesenchymal transition (EMT) and invasion in bladder cancer. Within the non-coding RNA regulatory network, OCT4-pg5 and circ_VANGL1 act as ceRNAs to sequester miR-145, promoting cisplatin resistance via the OCT4B/Wnt pathway and doxorubicin resistance through SOX4 activation, respectively. lncRNA TUG1 suppresses miR-145 to activate ZEB2, driving EMT and reducing radiotherapy sensitivity. Additionally, the TGFβ1/lncRNA-LET/NF90 axis impedes pri-miR-145 maturation, upregulating cancer stem cell (CSC) markers HMGA2 and KLF4, thereby exacerbating gemcitabine resistance. Collectively, miR-145 overcomes therapy resistance by targeting stemness maintenance, inflammatory signaling, and EMT pathways, though its efficacy is dynamically modulated by microenvironmental cues. TR4: Testicular orphan nuclear receptor 4; OCT4: Octamer-binding transcription factor 4; IKKα: Inhibitory Kappa B Kinase α; NF-κβ: Nuclear factor kappa-β; COX-2: Cyclooxygenase-2; EMT: Epithelial-mesenchymal transition; TGF-β: Transforming growth factor-β; NF90: Nuclear factor 90; SOX4: SRY-Box Transcription Factor 4; TGFBR2: Transforming Growth Factor Beta Receptor 2; ZEB2: Zinc Finger E-Box Binding Homeobox 2.

Chemotherapy and radiotherapy are fundamental treatments for cancer. Although patients may initially exhibit a favorable response, cancer cells often develop resistance over time, resulting in decreased efficacy or recurrence. Consequently, overcoming drug resistance remains a pivotal challenge in cancer research. Recent studies indicate that miR-145 plays a significant role in the resistance of urological tumors to chemotherapy and radiotherapy, suggesting that miR-145 mimics or inhibitors could enhance the efficacy of these treatments. TTM is widely utilized in China for treating conditions such as hypertension, headaches, and inflammation (Qian et al., 2020). Trillin, an extract from TTM, has garnered considerable attention due to its documented anticancer properties. Previous studies demonstrate that Trillin inhibits autophagy, induces apoptosis in hepatocellular carcinoma cells, reduces invasion, and impedes tumor growth (Zhan et al., 2024). Furthermore, Trillin significantly inhibits cell viability and proliferation in castration-resistant prostate cancer (CRPC) cell lines (DU145 and PC3) and mouse models, with its mechanism of action closely associated with the regulation of miR-145-5p. Trillin enhances the expression of miR-145-5p in CRPC cells and downregulates MAP3K11 while inhibiting inflammatory pathways through miR-145-5p. Specifically, Trillin downregulates MAP3K11 and inhibits the inflammatory pathway molecules COX-2 and NF-κB, thereby exerting its anticancer effects in prostate cancer (Wang et al., 2025). The low expression of miR-145 in prostate cancer leads to the upregulation of its direct target, OCT4. As a stem cell-associated transcription factor, OCT4 promotes the survival and migratory capabilities of tumor cells through the activation of downstream signaling pathways, such as the cell cycle regulatory protein cyclin D1, thereby enhancing chemotherapy resistance. Experimental evidence indicates that the overexpression of miR-145 significantly inhibits OCT4 protein levels by specifically binding to the 3′-UTR region of OCT4, as verified by luciferase reporter assays. This interaction reduces the resistance of cancer cells to chemotherapeutic agents, such as docetaxel, while also inhibiting cell proliferation and migration (Zhu et al., 2021). Furthermore, one study demonstrated that the pseudogene OCT4-pg5 competes with miR-145-5p through a ceRNA mechanism, which disrupts the inhibitory effect of miR-145-5p on OCT4B, consequently leading to the upregulation of OCT4B expression. This regulatory network enhances the expression of EMT-related molecules, including MMP2/9 and ZEB1/2, via the activation of the Wnt/β-catenin signaling pathway. Additionally, it decreases apoptosis and increases the proportion of G1-phase cells, ultimately resulting in a reduced sensitivity of bladder cancer cells to chemotherapeutic agents, such as cisplatin (Zhou et al., 2024) (Figure 4).

Celecoxib directly targets and inhibits the protein expression of TGF-β receptor 2 (TGFBR2) by upregulating the expression of miR-145, thereby blocking the activation of the TGF-β signaling pathway. Through this mechanism, celecoxib exhibits synergistic inhibitory effects on the migration and invasion of bladder cancer cells when combined with miR-145 mimics. This inhibition of EMT may reduce chemoresistance by diminishing the invasive phenotype of tumor cells (Liu et al., 2019). Circ_VANGL1 is highly expressed in bladder cancer tissues and cells, functioning as a “molecular sponge” by directly binding to miR-145-5p. This interaction releases the inhibitory effect of miR-145-5p on SOX4, thereby activating the SOX4 signaling pathway. Consequently, miR-145-5p downregulates SOX4 expression, which inhibits bladder cancer cell proliferation, promotes apoptosis, and enhances sensitivity to chemotherapeutic agents such as adriamycin (Zhu and Zhang, 2021). Additionally, TGFβ1 inhibits lncRNA-LET expression by activating the TGFβ/SMAD signaling pathway and directly binding to the SMAD-binding element (SBE) in the lncRNA-LET promoter. This reduces the negative regulatory effect of lncRNA-LET on NF90 protein stability. Elevated NF90 levels lead to a decrease in mature miR-145 levels by binding to and inhibiting the processing of pri-miR-145, thereby deregulating the inhibitory effects of miR-145 on cancer stem cell (CSC) markers such as HMGA2 and KLF4. This process promotes the enrichment of bladder cancer stem cells and the development of EMT, ultimately enhancing the resistance of tumor cells to chemotherapeutic agents such as gemcitabine (Zhuang et al., 2017). It has been demonstrated that LncRNA TUG1 reduces the expression of miR-145, with ZEB2 identified as a direct target of miR-145. ZEB2 expression has been reported across various tumors, including bladder cancer (Lee et al., 2014; Prislei et al., 2015). Recent studies have highlighted the close association of ZEB2 with EMT (Huang et al., 2015; Yang et al., 2015), indicating that ZEB2 plays a pivotal role in cancer development and progression. Notably, ZEB2 has been shown to induce the deletion of the epithelial marker E-cadherin, thereby disrupting intercellular adhesion. Furthermore, ZEB2 is capable of upregulating mesenchymal markers such as N-cadherin and vimentin (Kurashige et al., 2012; Bindels et al., 2006). These findings suggest that ZEB2 may serve as a crucial mediator of EMT in the context of cancer development. Interestingly, ZEB2 also protects bladder cancer cells from radiation-induced apoptosis. The data indicate that TUG1 enhances ZEB2 expression by negatively regulating miR-145. The miR-145/ZEB2 axis mediates the role of TUG1 in EMT and radiation resistance (Tan et al., 2015).

## Conclusion and outlook

MiR-145 is a small non-coding RNA molecule recognized as a potent anticancer agent across various tumors, particularly urological tumors, which are known for their aggressive nature and poor prognosis. As a critical oncogenic molecule, miR-145 inhibits the proliferation, invasion, and metastasis of prostate, bladder, and renal cancers while inducing apoptosis in tumor cells by targeting and regulating oncogenic signaling pathways, such as PLD5, SWAP70, and STAT3. This suggests its potential role in modulating various cellular processes involved in oncogenesis and inhibiting tumor progression. Existing studies mainly support the oncogenic role of miR-145 in urological tumours, but a few evidences suggest that it may exert a pro-tumourigenic function in the metastatic stage of bladder cancer by down-regulating targets such as NDRG1 (Jiang et al., 2017; Heishima et al., 2023). This stage-dependent functional switch suggests that the biological role of miR-145 is highly complex and may be influenced by the tumour microenvironment, epigenetic modifications and synergistic signalling pathways. In the future, it is necessary to further validate its pro-tumour mechanism in specific clinical scenarios and explore the potential risks of its use as a therapeutic target. Chemotherapy and radiotherapy are primary treatment strategies for patients with urological tumors; however, treatment resistance often limits their effectiveness in improving prognosis. Cancers are complex dynamic systems that develop drug-resistant strategies upon the application of therapies. Consequently, residual tumor cells in some patients may become resistant to treatment, resulting in recurrence and metastasis. Resistance to chemotherapy and radiotherapy presents a significant challenge in contemporary cancer research. Therefore, developing therapeutic agents that either block the downregulation or enhance the expression of miR-145 may offer a promising strategy for combating urological tumors. Current evidence reveals stark divergence in miR-145s targets across urologic tumors (e.g., SOX11 vs. TAGLN2), underscoring the need for cross-cancer studies to distinguish conserved from context-dependent mechanisms. Integrating multi-omics data and advanced models (e.g., organoids) could map miR-145s interactome, identifying universal vulnerabilities while guiding tissue-specific therapeutic strategies. Despite these challenges, miRNAs may provide significant advantages in the detection, prediction, and treatment of cancer. This article reviews the role of miR-145 in tumorigenesis and the progression of urological tumors. In conclusion, miR-145 may serve as a potential biomarker and therapeutic target for urological tumors, potentially paving the way for advancements in prognostic prediction, early diagnosis, and assessment of patients’ responses to therapy. However, further research is required to fully elucidate the interactions between miR-145 and urological tumors before its application in therapeutic contexts.
